# gGATLDA: lncRNA-disease association prediction based on graph-level graph attention network

**DOI:** 10.1186/s12859-021-04548-z

**Published:** 2022-01-04

**Authors:** Li Wang, Cheng Zhong

**Affiliations:** 1 School of Computer Science and Engineering, South China University of Technology, Guangzhou, China; 2 School of Computer, Electronics and Information, Guangxi University, Nanning, China; 3 Key Laboratory of Parallel and Distributed Computing in Guangxi Colleges and Universities, Guangxi University, Nanning, China

**Keywords:** lncRNA-disease association prediction, Graph attention network, Gaussian interaction profile kernel similarity of lncRNAs, Disease similarity based on gene–gene interaction network

## Abstract

**Background:**

Long non-coding RNAs (lncRNAs) are related to human diseases by regulating gene expression. Identifying lncRNA-disease associations (LDAs) will contribute to diagnose, treatment, and prognosis of diseases. However, the identification of LDAs by the biological experiments is time-consuming, costly and inefficient. Therefore, the development of efficient and high-accuracy computational methods for predicting LDAs is of great significance.

**Results:**

In this paper, we propose a novel computational method (gGATLDA) to predict LDAs based on graph-level graph attention network. Firstly, we extract the enclosing subgraphs of each lncRNA-disease pair. Secondly, we construct the feature vectors by integrating lncRNA similarity and disease similarity as node attributes in subgraphs. Finally, we train a graph neural network (GNN) model by feeding the subgraphs and feature vectors to it, and use the trained GNN model to predict lncRNA-disease potential association scores. The experimental results show that our method can achieve higher area under the receiver operation characteristic curve (AUC), area under the precision recall curve (AUPR), accuracy and F1-Score than the state-of-the-art methods in five fold cross-validation. Case studies show that our method can effectively identify lncRNAs associated with breast cancer, gastric cancer, prostate cancer, and renal cancer.

**Conclusion:**

The experimental results indicate that our method is a useful approach for predicting potential LDAs.

## Introduction

Long non-coding RNAs (lncRNAs) is a kind of non-protein-coding RNA, which has over 200 nucleotides [1]. More and more researches have indicated that the mutations and dysregulations of lncRNAs are closely related to the development and progression of various human complex diseases, including cancer [2]. For example, the down-regulation of H19 significantly decreased breast cancer and lung cancer cell clonogenicity and anchorage- independent growth [3]. BCYRN1 was increased in non-small cell lung cancer (NSCLC), and its downregulated expression could suppress NSCLC cell proliferation and cell cycle progression by inhibiting the Wnt/βcatenin pathway [4]. MALAT-1 was highly expressed in NSCLC [5]. LncRNA-IUR family was a key negative regulator of Bcr-Abl- induced tumorigenesis. LncRNA-IUR-5 suppressed Bcr-Abl-mediated tumorigenesis by negatively regulating STAT5-mediated expression of CD71 [6]. HOTAIR played a carcinogenic role in different cancers, including breast cancer, gastric cancer, colorectal cancer and cervical cancer cell [7]. Preclinical studies indicated that LncRNA-SARCC could attenuate RCC cell invasion, migration and proliferation in vitro and in vivo [8]. The specific HOTAIRM1 cytoplasmicisoform HM1-3 was downregulated in over 90% of clear cell renal cell carcinomas (ccRCCs) [9].  Therefore, the identification of disease-related lncRNAs will help to understand human complex disease mechanism, disease diagnosis, treatment, prognosis and prevention at lncRNA level.

In recent years, the experimentally supported lncRNA-disease associations are gradually increasing, and these association data have been collected into several databases such as NONCODE [10], LncRNADisease [11], and Lnc2Cancer [12]. However, the known lncRNA-disease associations still involve small part of lncRNA-disease pairs. Due to the biological experiments are expensive and time-consuming, it is very necessary to develop effective and accurate computational method to identify the potential lncRNA-disease associations, which provide the basis for further biological experimental verification.

The existing LDAs prediction methods can be mainly categorized into the network-based methods, machine learning-based methods and matrix factorization-based methods.

The network-based methods construct global heterogeneous network by integrating known LDAs, disease similarities, and lncRNA similarities, and use random walk to identify potential LDAs [13, 14]. Sun et al. [15] proposed a novel LDAs prediction model based on a random walk on an lncRNA functional similarity network, called RWRlncD, to infer potential human LDAs. The limitation of the method was not applicable for lncRNAs that did not have any known associated diseases. Chen et al. [16] developed the prediction model KATZLDA using KATZ measure to predict potential lncRNA-disease association on the heterogeneous network. Huang et al. [17] developed an improved lncRNA functional similarity calculation model called ILNCSIM to improve prediction performance. Hu et al. [18] developed a bi-random walks algorithm BiWalkLDA to predict the LDAs. The bi-random walk referred that the two scores was obtained by performing random walk on disease similarity network and lncRNA similarity network respectively, and then the mean of two scores was used as the prediction result. Li et al. [19] proposed a target convergence set based LDAs prediction method, called TCSRWRLD. TCSRWRLD would establish a node set called Target Convergence Set (TCS) for each lncRNA/disease node in the constructed heterogeneous lncRNA-disease network, and an improved random walk with restart (RWR) was implemented on the heterogeneous lncRNA-disease network to infer potential LDAs. TCSRWRLD introduced the concept of TCS, which could effectively accelerate convergence of the algorithm. In order to improve prediction accuracy, some researchers integrated other biological information except lncRNA and disease, such as miRNAs and protein [20, 21]. Fan et al. [22] developed a method called IDHI-MIRW to predict LDAs. IDHI-MIRW used RWR algorithm on different lncRNA/disease similarities network to obtain the lncRNA/disease topological similarity through the positive pointwise mutual information (PPMI). Then, IDHI-MIRW applied the RWR algorithm on heterogeneous network by integrating the lncRNA/disease topological similarity and the known LDAs to predict the LDAs.

With the application of machine learning and deep learning in Biology [23–25], some LDAs prediction methods using different machine learning have been proposed, such as Bayesian classifier based prediction method [26], random forest based prediction method [27], and normal Laplacian regularized least squares based prediction method [28]. Chen et al. [29] proposed a semi-supervised learning method called LRLSLDA to identify potential associations between lncRNAs and diseases by using Laplacian regularized least squares, which was the first computational model to predict LDAs. LRLSLDA calculated lncRNA similarities and disease similarities, and formulated two classifiers based on Laplacian Regularized Least Squares in the disease space and lncRNA space respectively, and combined these two classifiers into a single classifier to obtain final association probability between disease and lncRNA. Xie et al. [30] presented a similarity kernel fusion method to predict LDAs, called SKF-LDA, which also used a normal Laplacian regularized least-squares method. SKF-LDA selected more appropriate fusion method to integrate more biological knowledge to obtain more accurate prediction results. The fusion method built the refined similarity matrices by a neighbor-based constraint and iteration over the similarity matrices instead of a simply weighted addition.

Deep learning has been applied to various prediction problems in Biology [31–33]. Xuan et al. proposed different deep learning-based lncRNA-disease prediction models, such as CNNLDA [34], GCNLDA [35], CNNDLP [36] and LDAPred [37]. CNNLDA used a double convolution neural network based on attention mechanism. GCNLDA used a graph convolution neural network. CNNDLP used convolution neural network and convolution automatic encoder. LDAPred used convolutional neural network and information flow propagation. Wei et al. [38] proposed a predictor named iLncRNAdis-FB to identify new LDAs. The method constructed three-dimensional feature blocks of lncRNA-disease pairs by integrating six different biological data, and then used convolutional neural network to predict unknown LDAs. Wang et al. [39] developed a multi-label classification with deep forest to predict LDAs. The model implemented multi-label classification by multi-grained scanning and cascade forest. In the multi-grained scanning part, the corresponding transformed feature representation was classified according to different forests. In the cascade forest, layer-wise random forest was used to get more discriminative representations. Yang et al. [40] proposed a bidirectional generative adversarial network model called BiGAN, which consisted of an encoder, a generator, and a discriminator. The encoder and generator were used to learn high-level features, the discriminator was used to predict LDAs.

At present, matrix factorization has been applied to identify potential LDAs [41–46]. Fu et al. [47] developed a matrix factorization based prediction model MFLDA. MFLDA fused the data sources by assigning different weights and decomposed the heterogeneous data sources into low-rank matrices by matrix tri-factorization. Lu et al. [48] proposed a LDA prediction method called SIMCLDA based on the inductive matrix completion. The method extracted primary feature vectors from lncRNA similarity and disease similarity by principle components analysis (PCA) respectively, and calculated the interaction profile between a new lncRNA and its neighbors, and completed the association matrix with inductive matrix completion using primary feature vectors and constructed interaction profiles. Compared with traditional matrix factorization-based prediction methods, deep learning based prediction methods can capture non-linear relationship between lncRNAs and diseases. Therefore, some researchers combined matrix factorization with deep learning to improve the performance of predicting LDAs [49, 50].

Recently, GNNs including graph convolution network and graph attention network have been applied in Bioinformatics [51–53]. Fan et al. [54] proposed a novel computational method GCRFLDA based on the graph convolutional matrix completion. The GCRFLDA integrated conditional random field (CRF) and attention mechanism into the encoder layer to learn the embedding of nodes, and scored potential lncRNA-disease associations. To improve prediction performance, we propose a novel method for predicting potential LDAs based on graph-level graph attention network. The main contributions of this paper are summarized as follows:

1. We propose a new disease similarity calculation based on gene–gene interaction network.
2. We propose a novel lncRNA-disease associations prediction method based on graph-level graph attention network.
3. The experimental results show that our method is superior to other state-of-the-art methods in evaluation metrics such as AUC, AUPR, F1-Score, recall, precision and accuracy.

The remainder of this paper is organized as follows: Section “Results” shows experimental results. Section “Conclusion” concludes the paper. Section “Datasets and methods” describes our proposed method in detail.

## Results

### Experimental setting

In our study, five fold cross-validation (CV) is conducted on the experiments to evaluate the prediction performance of our method and other methods. Three cross-validation settings are as follows:

1. CVP (cross-validation based on the LDA pairs): We randomly partition all experimentally verified LDA pairs into five subsets. In each fold, one subset is used as test set and the other four subsets are used as the training set. The previous training set and test set are positive samples. The unknown lncRNA-disease pairs with the same number of positive samples are randomly selected as negative samples.
2. CVL (cross-validation based on the lncRNAs): We randomly select 20% rows (i.e. lncRNAs) as testing set. The remaining 80% rows (i.e. lncRNAs) are used as training set.
3. CVD (cross-validation based on the diseases): We randomly select 20% columns (i.e. diseases) as testing set. The remaining 80% columns (i.e. diseases) are used as training set.

The CVL and CVD methods are designed to evaluate the capability of predicting LDAs for new lncRNAs and new diseases. Each cross-validation is repeatedly conducted for 10 times, and the average of 10 experimental results is used for final result.

### Comparisons with existing works

We compare our method gGATLDA with five state-of-the-art LDAs prediction methods: BiWalkLDA [18], MFLDA [47], SIMCLDA [48], BiGAN [40] and GCRFLDA [54]. BiWalkLDA performed bi-random walks on lncRNA- disease network integrating interaction profile and gene ontology information to predict LDAs. MFLDA fused different heterogeneous data and predicted new associations using matrix factorization. SIMCLDA was a method for predicting potential LDAs based on inductive matrix completion. BiGAN was an lncRNA-disease association prediction method based on bidirectional generative adversarial network. GCRFLDA was a prediction method based on the graph convolutional matrix completion. We implemented the experimental codes based on deep learning framework Pytorch.

**Fig. 1:**
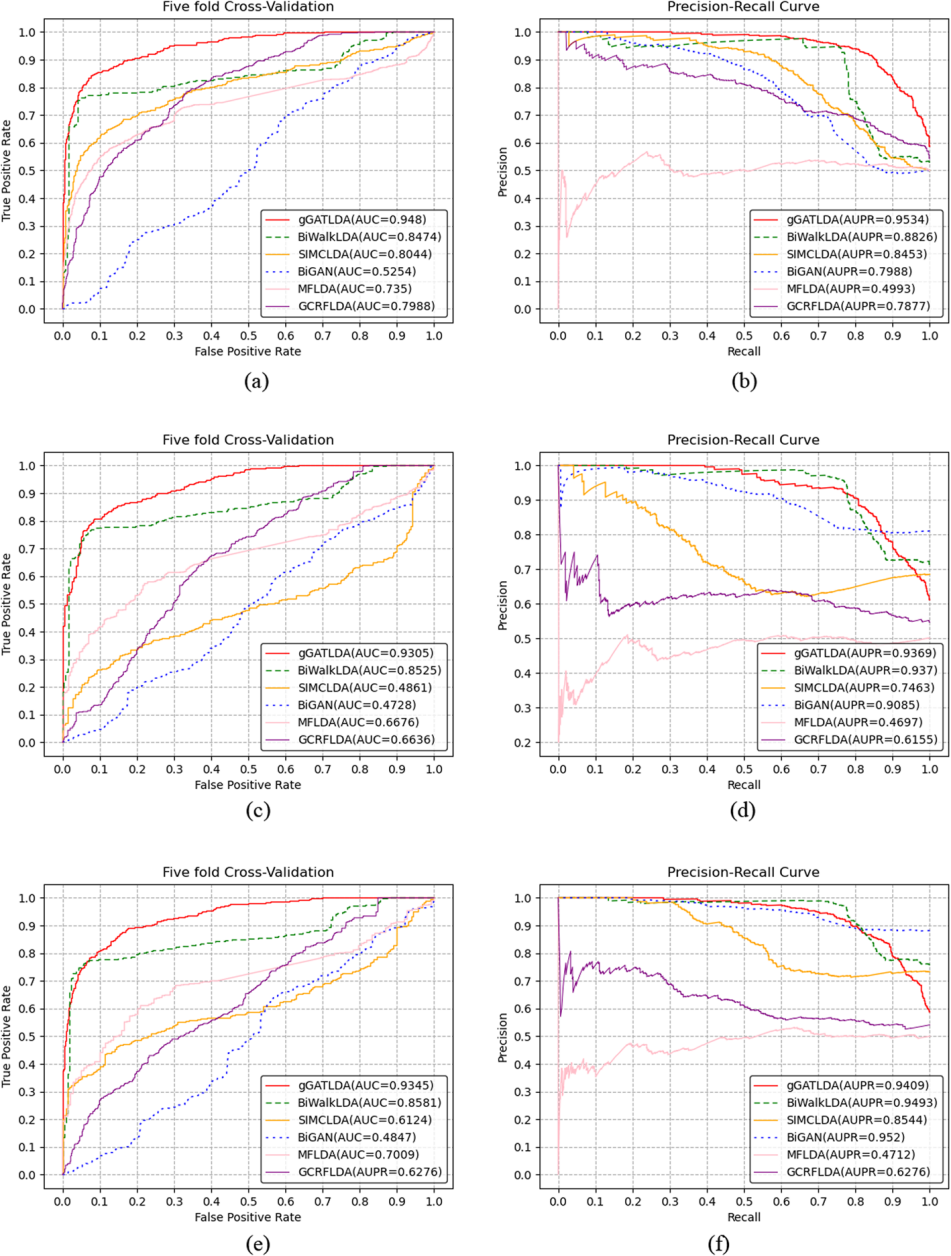
Performance comparison of predicting methods under the setting CVP, CVL and CVD on Dataset 1. **a**–**b** Performance of all methods based on the CVP cross-validation settings. **c**–**d** Performance of all methods based on the CVL cross-validation settings. **e**–**f** Performance of all methods based on the CVD cross-validation settings

**Fig. 2:**
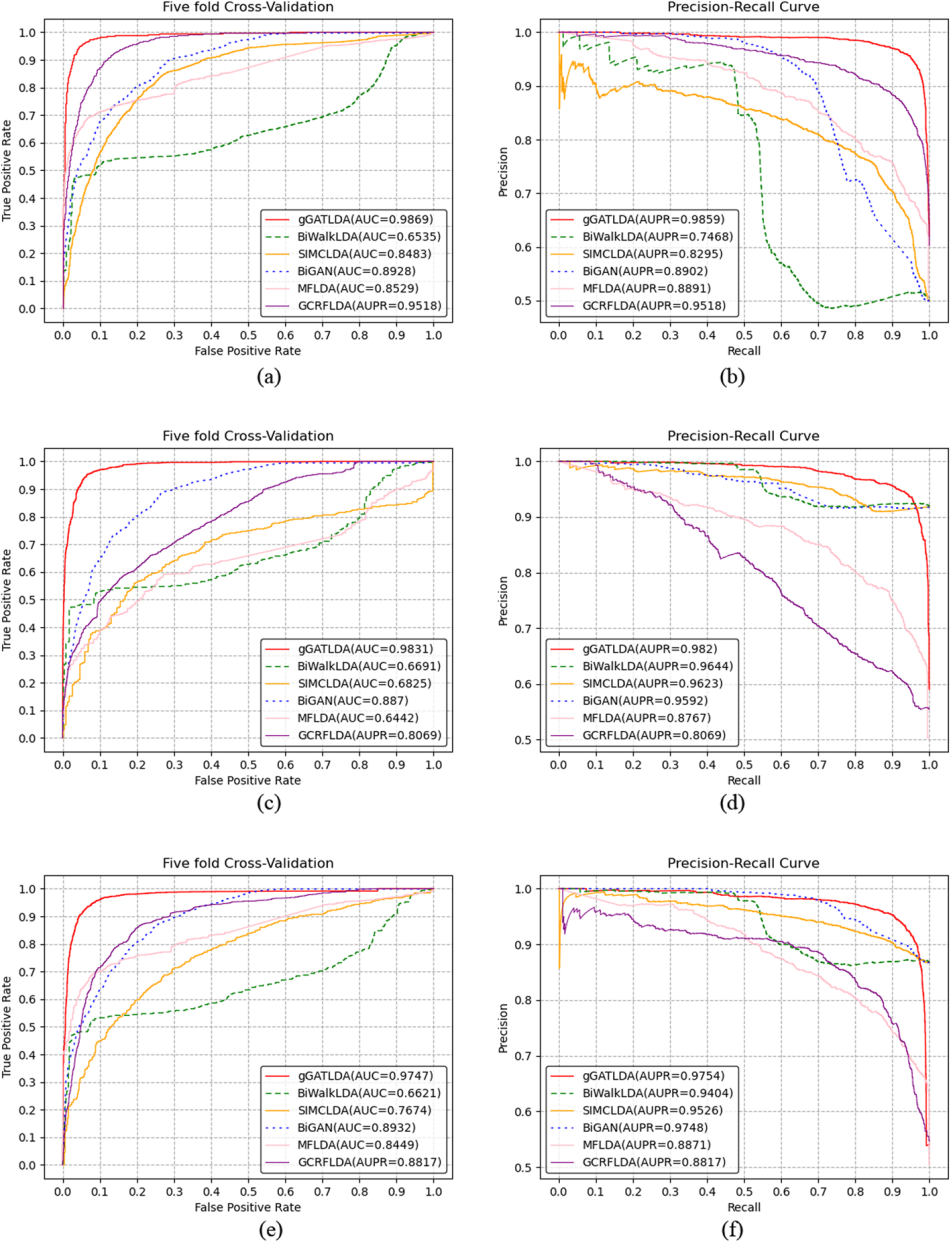
Performance comparison of predicting methods under the setting CVP, CVL and CVD on Dataset 2. **a**–**b** Performance of all methods based on the CVP cross-validation settings. **c**–**d** Performance of all methods based on the CVL cross-validation settings. **e**–**f** Performance of all methods based on the CVD cross-validation settings

**Fig. 3:**
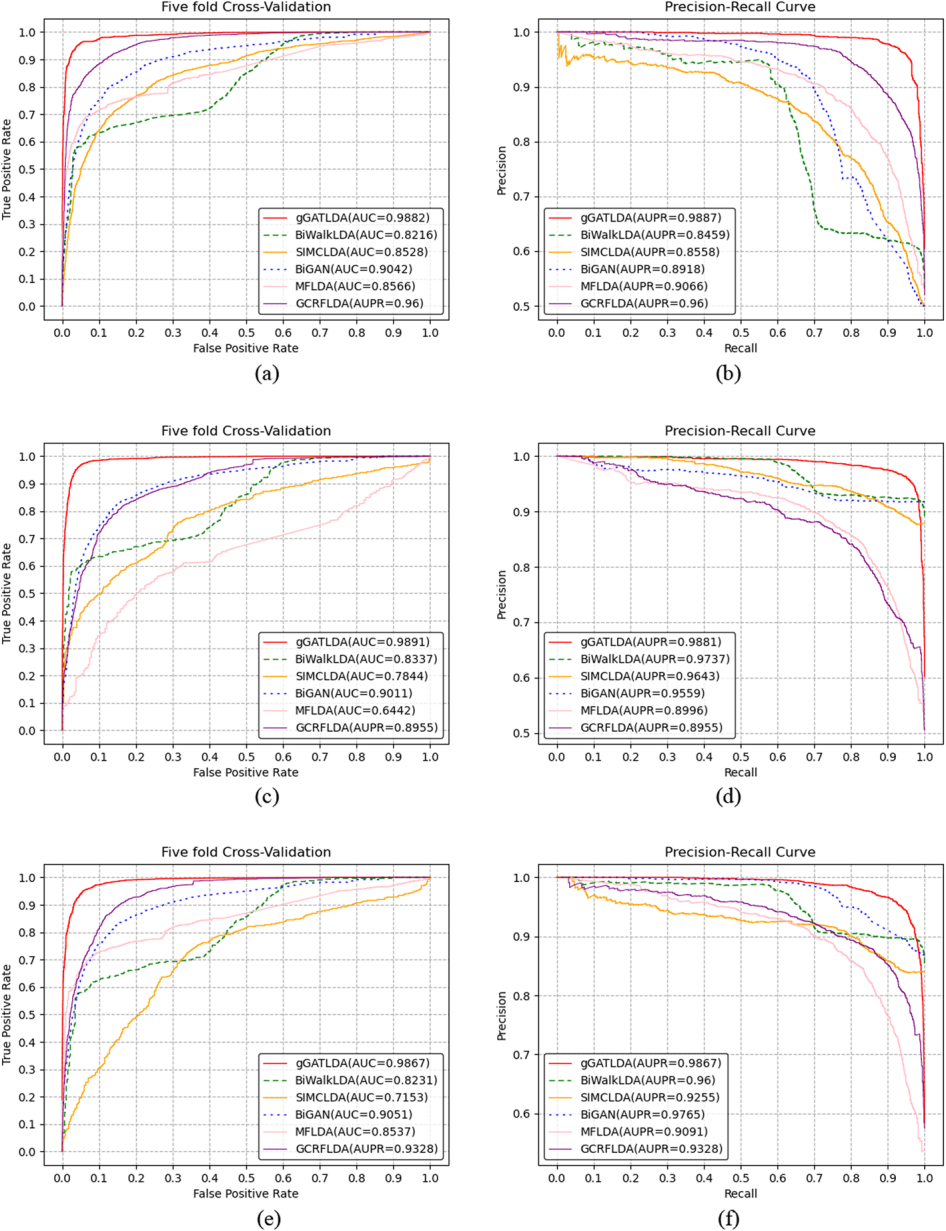
Performance comparison of predicting methods under the setting CVP, CVL and CVD on Dataset 3. **a**–**b** Performance of all methods based on the CVP cross-validation settings. **c**–**d** Performance of all methods based on the CVL cross-validation settings. **e**–**f** Performance of all methods based on the CVD cross-validation settings

We evaluate our method gGATLDA and other five methods in terms of AUC and AUPR. We select the three different benchmark datasets including different numbers of known LDAs. Dataset1 contained only 621 LDAs, Dataset2 contained 2697 LDAs, and Dataset3 contained 3207 LDAs obtained by merging the Dataset1 and Dataset2. Under three cross-validation settings (CVP, CVL, and CVD), we conduct the experiments on three benchmark datasets respectively. The experimental results are shown in Figs. 1, 2 and 3.

As can be seen from Figs. 1, 2 and 3, our method gGATLDA can achieve the best prediction performance. For example, as shown in Fig. 1a, gGATLDA achieve the highest average AUC of 0.948 under the setting CVP, which is 11.9% higher than the secondly best BiWalkLDA. Figure 1b show that GATLDA achieve a higher precision with respect to the other five methods for any given recall value. As shown in Fig. 1c, under the CVL and CVD, the AUC and AUPR value of gGATLDA are highest respectively.

On different benchmark datasets, the prediction performance of each method is different. As can be seen from Figs. 1, 2 and 3, the prediction performance of our method and SIMCLDA is more stable, and the AUC and AUPR value of our method gGATLDA have higher than that of SIMCLDA on three different datasets. On Dataset1, our method has highest AUC and AUPR. On Dataset2 , the AUC of our method are 3.7%, 10.2%, 15.7%, 16.3% and 55.8% higher than the other five methods GCRFLDA, BiGAN, MFLDA, SIMCLDA and BiWalkLDA respectively. On Dataset3, the AUC of our method are 2.9%, 9.3%, 15.4%, 15.9% and 20.3% higher than the other five methods GCRFLDA, BiGAN, MFLDA, SIMCLDA and BiWalkLDA respectively. The other four methods, such as BiWalkLDA, MFLDA, BiGAN and GCRFLDA, have different prediction performance on different datasets. For example, the AUC of BiGAN is only 0.4847 on Dataset 1, but its AUC value is 0.9042 on Dataset 3 (the latter is about twice the former). BiGAN and GCRFLDA both obtain the highest AUC and AUPR on Dataset 3, which show that the two methods are more suitable for Dataset 3. MFLDA has the lowest values of AUPR on Dataset 1 under the CVP, CVL, and CVD cross-validation settings, which are 0.4993, 0.4697 and 0.4712 respectively. However, on Datasets 2 and 3, the AUPR of MFLDA achieved 0.8891 and 0.9066 respectively. These indicates that MFLDA is sensitive to different datasets. BiWalkLDA perform best on Dataset 1, and perform worst on Dataset 2. Therefore, BiWalkLDA is also sensitive to different datasets.

Different cross validation settings have different influence on the prediction performance of different methods. Under three cross-validation settings, the ROC curve and PR curve of our method are essentially the same. However, the prediction performance of other five methods greatly differs under different cross validation settings. For example, on Dataset1, the AUC of SIMCLDA are 0.8044, 0.4861 and 0.6124 under the CVP, CVL and CVD respectively, while on the Dataset 2, its AUC are 0.8483, 0.6825 and 0.7674 respectively.

In addition to AUC and AUPR, we utilize other evaluation metrics including the F1-score, accuracy, precision and recall to evaluate the performance of our model. Under CVP setting, the experimental results on the three datasets are shown in Tables 1, 2 and 3. As shown in Table 1, on Dataset 1, our method obtain the highest value of all evaluation metrics such as AUC, AUPR, accuracy, F1-score, recall and precision, which show that gGATLDA can achieve better prediction results on Dataset1. Considering that the number of known associations in Dataset 2 is relatively more, we also compare the different performance evaluation metrics of the six prediction methods on Dataset 2. Table 2 show that gGATLDA obtain the best prediction performance. For example, the accuracy of gGATLDA, BiWalkLDA, SIMCLDA, MFLDA, BiGAN and GCRFLDA are 0.9395, 0.4930, 0.7549, 0.7698, 0.8016 and 0.8859 respectively. The F1-score of our method is 0.0661 higher than that of the second ranked method GCRFLDA. Table 3 show that evaluation metrics (AUC, AUPR, F1-score and recall) of our method are higher than other five prediction methods on Dataset3. However, the value of the accuracy and precision are lower than GCRFLDA.

**Table 1:** Experiment results of six methods on Dataset1 under CVP setting

	gGATLDA	BiWalkLDA	SIMCLDA	MFLDA	BiGAN	GCRFLDA
AUC	**0.9442** $\pm$ **0.0025**	0.8435 $\pm$ 0.0028	0.7836 $\pm$ 0.0113	0.7223 $\pm$ 0.0118	0.5246 $\pm$ 0.0475	0.8120 $\pm$ 0.0174
Precision	**0.8124** $\pm$ **0.0346**	0.7538 $\pm$ 0.0325	0.6822 $\pm$ 0.0832	0.6928 $\pm$ 0.1351	0.4972 $\pm$ 0.0555	0.7273 $\pm$ 0.0197
Recall	**0.9029** $\pm$ **0.0276**	0.7968 $\pm$ 0.0135	0.7591 $\pm$ 0.0861	0.6705 $\pm$ 0.1342	0.5025 $\pm$ 0.0759	0.7025 $\pm$ 0.0473
AUPR	**0.9493** $\pm$ **0.0022**	0.8727 $\pm$ 0.0079	0.8203 $\pm$ 0.0125	0.7895 $\pm$ 0.0100	0.5029 $\pm$ 0.0422	0.7806 $\pm$ 0.0236
Accuracy	**0.8455** $\pm$ **0.0150**	0.7768 $\pm$ 0.0216	0.6866 $\pm$ 0.0546	0.6432 $\pm$ 0.0941	0.4992 $\pm$ 0.0551	0.7473 $\pm$ 0.0153
F1-Score	**0.8541** $\pm$ **0.0093**	0.7740 $\pm$ 0.0128	0.7087 $\pm$ 0.0200	0.6552 $\pm$ 0.0205	0.4995 $\pm$ 0.0651	0.7127 $\pm$ 0.0323

The best results in each row are represented in bold

**Table 2:** Experiment results of six methods on Dataset2 under CVP setting

	gGATLDA	BiWalkLDA	SIMCLDA	MFLDA	BiGAN	GCRFLDA
AUC	**0.9870 ± 0.0024**	0.6499 ± 0.0022	0.8433 ± 0.0035	0.8270 ± 0.0033	0.8932 ± 0.0118	0.9548 $\pm$ 0.0055
Precision	**0.9098 ± 0.0136**	0.4958 ± 0.0040	0.6979 ± 0.0114	0.9261 ± 0.0368	0.8031 ± 0.0129	0.8840 $\pm$ 0.0063
Recall	**0.9759 ± 0.0068**	0.8466 ± 0.0264	0.8997 ± 0.0103	0.5905 ± 0.0646	0.7990 ± 0.0443	0.8689 $\pm$ 0.0208
AUPR	**0.9864 ± 0.0025**	0.7419 ± 0.0036	0.8824 ± 0.0053	0.8720 ± 0.0027	0.8857 ± 0.0200	0.9512 $\pm$ 0.0088
Accuracy	**0.9395 ± 0.0083**	0.4930 ± 0.0065	0.7549 ± 0.0080	0.7698 ± 0.0166	0.8016 ± 0.0214	0.8859 $\pm$ 0.0077
F1-Score	**0.9416 ± 0.0076**	0.6253 ± 0.0104	0.7859 ± 0.0041	0.7174 ± 0.0358	0.8005 ± 0.0261	0.8755 $\pm$ 0.0125

The best results in each row are represented in bold

**Table 3:** Experiment results of six methods on Dataset3 under CVP setting

	gGATLDA	BiWalkLDA	SIMCLDA	MFLDA	BiGAN	GCRFLDA
AUC	**0.9888 ± 0.0065**	0.8185 ± 0.0024	0.8465 ± 0.0030	0.8478 ± 0.0048	0.9045 ± 0.0185	0.9583 $\pm$ 0.0055
Precision	0.7980 ± 0.0367	0.6370 ± 0.0033	0.7247 ± 0.0142	0.8667 ± 0.1310	0.6572 ± 0.0073	**0.9020** $\pm$ **0.0052**
Recall	**0.9913 ± 0.0078**	0.7297 ± 0.0121	0.8475 ± 0.0162	0.6942 ± 0.1089	0.9495 ± 0.0132	0.8632 $\pm$ 00,202
AUPR	**0.9890 ± 0.0060**	0.8416 ± 0.0031	0.8450 ± 0.0053	0.8860 ± 0.0032	0.9058 ± 0.0192	0.9548 $\pm$ 0.0090
Accuracy	0.8670 ± 0.0271	0.6568 ± 0.0032	0.7623 ± 0.0065	0.7652 ± 0.0867	0.7270 ± 0.0088	**0.9103** $\pm$ **0.0044**
F1-Score	**0.8830 ± 0.0217**	0.6801 ± 0.0049	0.7810 ± 0.0022	0.7523 ± 0.0324	0.7767 ± 0.0068	0.8817 $\pm$ 0.0130

The best results in each row are represented in bold

In summary, our method outperforms the other five methods in six evaluation metrics such as AUC, AUPR, accuracy, F1-score, recall and precision.

### Comparison of two disease similarities

**Fig. 4:**
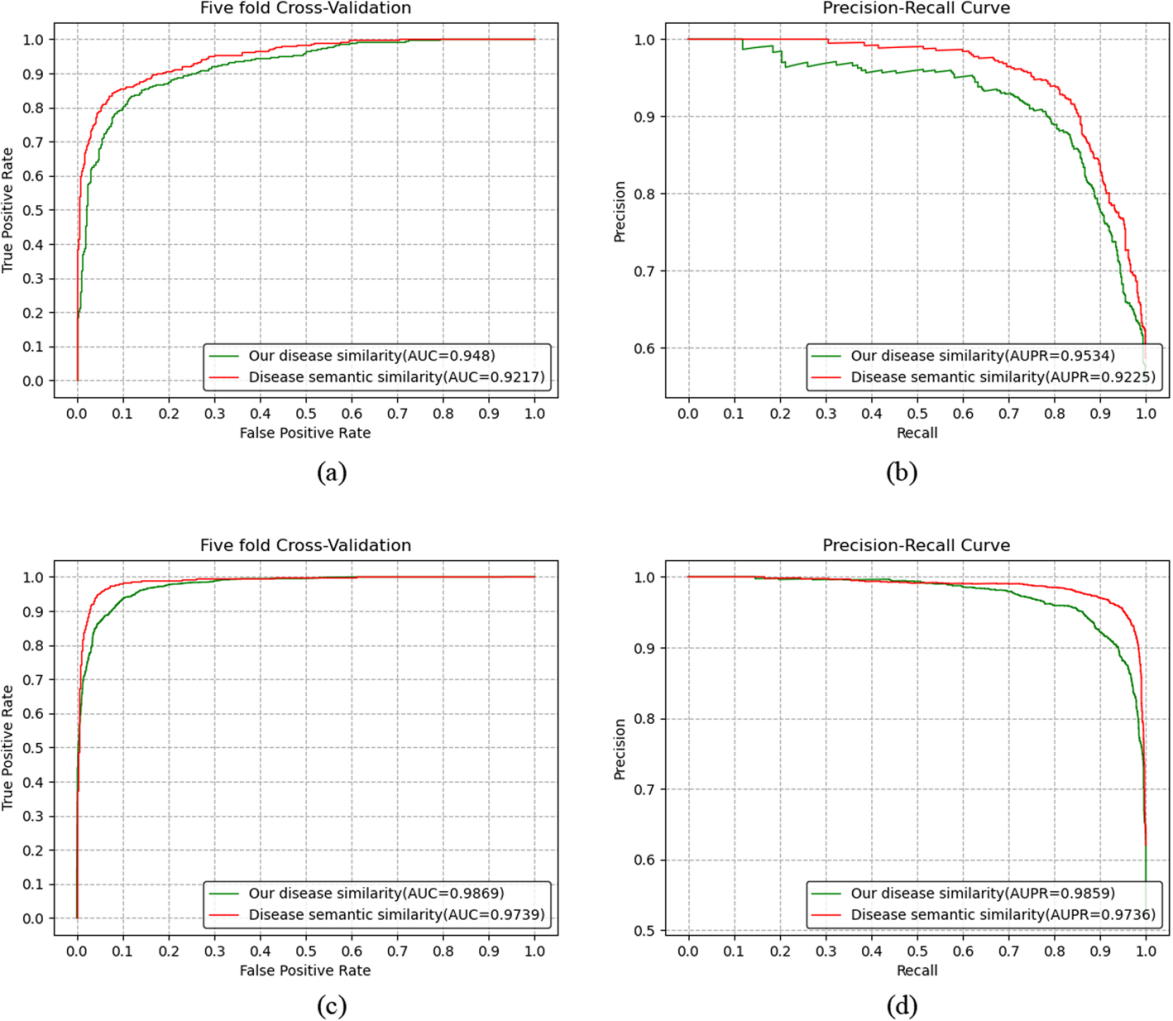
Performance comparison of predicting methods using different disease similarity. **a**–**b** For Dataset1, ROC curve and PR curve of predicting methods using different disease similarity. **c**–**d** For Dataset2, ROC curve and PR curve of predicting methods using different disease similarity

For the same model, different disease similarities have different effects on the performance of lncRNA-disease association prediction method. In the paper, we propose a new disease similarity calculation based on gene–gene interaction network. In order to evaluate the performance of our proposed disease similarity calculation, we compare it with disease semantic similarity using DAGs on Dataset1 and Dataset2 by evaluation of ROC curves, AUC values, PR curves and AUPR values in the five fold CV experiment. The experimental results are shown in Fig. 4. We can see from Fig. 4 that for the Dataset2, the AUC and AUPR values of our proposed disease similarity are higher than that of disease semantic similarity, and for the Dataset1, the prediction model using our proposed disease similarity also performed better than the prediction model using disease semantic similarity. It illustrates that the performance of the lncRNA-disease association prediction method using our proposed disease similarity can be improved.

### Influence of different number of hops on the accuracy of the model

GNN explores how to generate node embedding by aggregating neighborhood nodes, most of which are node-level embedding. GNN based on subgraph-level embedding can better learn the local structure of graph to improve performance. Weisfeiler-Lehman Neural Machine (WLNM) method proposed a solution to find the appropriate methods automatically, based on the extracted subgraphs in its neighborhood [55]. WLNM used high-order heuristics to achieve significant accuracy. However, high-order heuristics required a large number of hops that span the enclosing subgraphs to the global network, which would lead to additional computation time and memory. SEAL derived γ-decaying theory to infer that a small number of hops was enough to extract the high-order heuristics and achieved better accuracy than WLNM [56].

In this study, we focus on whether different hops are influence on the accuracy of the prediction model. We test the effects of different number of hops. We train our model using different number of hops respectively. Table 4 show that our model has little difference in performance evaluation metrics for enclosing subgraphs with different number of hops. However, when the number of hops increases, the number of nodes in the subgraph also increases, which will lead to memory and computational overhead. Based on comprehensive consideration, we choose 1-hop enclosing subgraphs in our experiment.

**Table 4:** Influence of different hops on the prediction model

	Dataset1			Dataset2		
	hop = 1	hop = 2	hop = 3	hop = 1	hop = 2	hop = 3
AUC	0.948	0.943	0.945	0.986	0.982	0.953
Precision	0.731	0.794	0.754	0.658	0.698	0.730
Recall	0.965	0.900	0.926	0.999	0.995	0.988
AUPR	0.953	0.948	0.951	0.985	0.983	0.950
Accuracy	0.799	0.825	0.800	0.732	0.777	0.802
F1-Score	0.830	0.838	0.824	0.791	0.819	0.837

### Parameter optimization

**Fig. 5:**
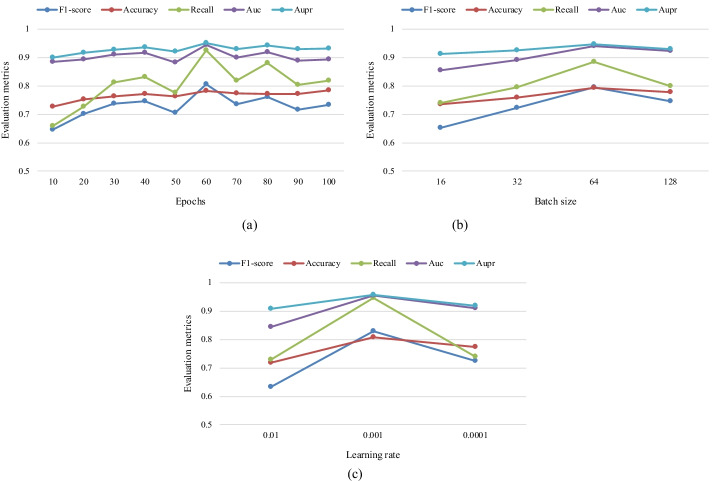
Hype-parameter optimization results for F1-score, accuracy, recall, AUC, AUPR. **a** Results comparing different epochs. **b** Results comparing different batch size. **c** Results comparing different learning rate

Different hyper-parameters will affect the prediction performance of gGATLDA. To obtain the best performance, we have tried a set of different hyper-parameters to find the best hyper-parameter for predicting lncRNA-disease associations. For the parameter dropout, we use the value suggested in most papers, i.e. 0.5. We perform a grid search to optimize three main hyper-parameters, namely, epochs from 10 to 100 with step 10, batch size with the values in {16, 32, 64, 128}, and learning rate with the values in {0.1, 0.01, 0.001}. We respectively tune each parameter combination to calculate the AUC, AUPR, F1-score, accuracy, recall of our model based on five fold cross validation. As shown in Fig. 5a, the four evaluation metrics including AUC, AUPR, F1-score and recall achieve the best results considering 60 as the number of epochs. From Fig. 5b and c, we can find that all evaluation metrics obtained the best performance when batch size is 64 and learning rate is 0.001. Finally, the optimal values of hyper-parameters such as learning rate, batch size, and number of training epochs is 0.001, 64 and 60 respectively.

### Case study

For further confirming the effectiveness of gGATLDA, we conduct case studies on four diseases, i.e., breast cancer, gastric cancer, prostate cancer, and renal cancer. All the known LDAs in Dataset1 are used as training samples, and other unknown associations are regarded as candidate associations for validation. For the investigated disease *d*, all lncRNAs unassociated with disease *d* are considered as candidate lncRNAs. We rank the candidate lncRNAs according to their predicted scores, and select the top 15 ones to verify whether associated with diseases based on two databases, namely, Lnc2Cancer and LncRNADisease v2.0. For those predicted results that are not been included in the Lnc2Cancer and LncRNADisease, we manually check in PubMed and list the supportive literatures. Moreover, in order to verify the effectiveness of our proposed disease similarity calculation method, we compare the two different disease similarities in the case studies. The validation results are shown in Tables 5, 6, 7 and 8. As can be seen from Tables 5–8 that in each case study, the verified proportion of the top 15 candidate lncRNAs obtained using our disease similarity is higher than that using disease semantic similarity.

Breast neoplasms is one of the most common female cancers. With the development of cancer research, lncRNAs have become an essential target for breast cancer prevention, diagnosis, and treatment. The top 15 predicted lncRNAs were experimentally verified by Lnc2Cancer, LncRNADisease v2.0, and published literatures (see Table 5). KCNQ1OT1 is found to be remarkably high expression in breast cancer tissues and cells, which promoted tumor growth in vivo by regulatingmiR-145/CCNE2 [57]. CCND1 is associated with cell cycle dysregulation in breast cancer [58]. CCND1 is a target of miR-142, and miR-142 inhibited proliferation of endometrial cancer cells by targeting CCND1 [59].

**Table 5:** Top 15 predicted lncRNAs associated with breast cancer

Disease similarity based on gene–gene interaction network			Disease similarity based on disease semantic		
Rank	lncRNA	Evidence	Rank	lncRNA	Evidence
1	KCNQ1OT1	Lnc2Cancer 3.0	1	TRAF3IP2-AS1	PMID: 30157476
2	UCA1	Lnc2Cancer 3.0	2	DLX6-AS1	Lnc2Cancer 3.0
3	MIAT	Lnc2Cancer 3.0	3	MINA	PMID: 30254753
4	MINA	PMID: 30254753	4	KCNQ1OT1	Lnc2Cancer 3.0
5	NPTN-IT1	Lnc2Cancer 3.0	5	NEAT1	Lnc2Cancer 3.0
6	LincRNA-p21	Lnc2Cancer 3.0	6	LincRNA-p21	Lnc2Cancer 3.0
7	IGF2-AS	PMID: 33175607	7	UCA1	Lnc2Cancer 3.0
8	DRAIC	LncRNADisease v2.0	8	SOX2-OT	Lnc2Cancer 3.0
9	NEAT1	Lnc2Cancer 3.0	9	NPTN-IT1	Lnc2Cancer 3.0
10	PCAT29	PMID: 32521844	10	HULC	Lnc2Cancer 3.0
11	HULC	Lnc2Cancer 3.0	11	CRNDE	LncRNADisease v2.0
12	CCND1	LncRNADisease v2.0	12	TUSC7	Lnc2Cancer 3.0
13	SPRY4-IT1	Lnc2Cancer 3.0	13	7SK	unconfirmed
14	SOX2-OT	Lnc2Cancer 3.0	14	WT1-AS	LncRNADisease v2.0
15	TUSC7	Lnc2Cancer 3.0	15	ESCCAL-1	unconfirmed

Gastric cancer is the fifth most common cancer and the third most common cause of cancer death globally. It is a molecularly and phenotypically highly heterogeneous disease. Multiple evidences demonstrate that lncRNAs play a vital role in gastric cancer resistance to chemotherapy reagents and targeted therapy drugs [60]. All top-15 candidate lncRNAs predicted by gGATLDA have confirmed to be associated with gastric cancer (see Table 6). DLX6-AS1 is over-expressed in gastric cancer tissues and cell lines, which regulate tumor growth and aerobic glycolysis in gastric cancer by targeting miR-4290 and PDK1 [61].

**Table 6:** Top 15 predicted lncRNAs associated with gastric cancer

Disease similarity based on gene–gene interaction network			Disease similarity based on disease semantic		
Rank	lncRNA	Evidence	Rank	lncRNA	Evidence
1	KCNQ1OT1	Lnc2Cancer 3.0	1	TRAF3IP2-AS1	PMID: 25370763
2	SOX2-OT	Lnc2Cancer 3.0	2	SOX2-OT	Lnc2Cancer 3.0
3	LincRNA-p21	Lnc2Cancer 3.0	3	DLX6-AS1	Lnc2Cancer 3.0
4	XIST	LncRNADisease v2.0	4	NEAT1	Lnc2Cancer 3.0
5	NPTN-IT1	Lnc2Cancer 3.0	5	MALAT1	Lnc2Cancer 3.0
6	MIAT	Lnc2Cancer 3.0	6	GAS5	Lnc2Cancer 3.0
7	DRAIC	Lnc2Cancer 3.0	7	XIST	LncRNADisease v2.0
8	MALAT1	Lnc2Cancer 3.0	8	LincRNA-p21	Lnc2Cancer 3.0
9	HULC	Lnc2Cancer 3.0	9	KCNQ1OT1	Lnc2Cancer 3.0
10	IGF2-AS	PMID: 31183590	10	NPTN-IT1	Lnc2Cancer 3.0
11	NEAT1	Lnc2Cancer 3.0	11	HULC	Lnc2Cancer 3.0
12	PCAT29	LncRNADisease v2.0	12	TUG1	Lnc2Cancer 3.0
13	AIR	Lnc2Cancer 3.0	13	MIAT	Lnc2Cancer 3.0
14	GAS5	Lnc2Cancer 3.0	14	DRAIC	Lnc2Cancer 3.0
15	TUG1	Lnc2Cancer 3.0	15	SRA1	unconfirmed

Prostate cancer is the most common malignancy in male around the world. For identifying a novel bio-labeling for early prediction and treatment in prostate cancer, it is urgently needed that identifying LDAs. We have confirmed 14 of the top-15 candidate lncRNAs to be association with prostate cancer by Lnc2Cancer, LncRNADisease, and published literatures (see Table 7). LncRNA MEG3 has a downregulated in prostate cancer and impact on the abilities of cell proliferation, migration and invasion, and cell apoptosis rate [62]. The candidate lncRNA TRAF3IP2-AS1 has no experimental evidence to prove that it is related to prostate cancer.

**Table 7:** Top 15 predicted lncRNAs associated with prostate cancer

Disease similarity based on gene–gene interaction network			Disease similarity based on disease semantic		
Rank	lncRNA	Evidence	Rank	lncRNA	Evidence
1	H19	LncRNADisease v2.0	1	TRAF3IP2-AS1	unconfirmed
2	MALAT1	Lnc2Cancer 3.0	2	DLX6-AS1	PMID: 33035382
3	TRAF3IP2-AS1	unconfirmed	3	SNHG11	Lnc2Cancer 3.0
4	PVT1	Lnc2Cancer 3.0	4	H19	LncRNADisease v2.0
5	MEG3	Lnc2Cancer 3.0	5	IGF2-AS	Lnc2Cancer 3.0
6	XIST	Lnc2Cancer 3.0	6	TERC	LncRNADisease v2.0
7	CDKN2B-AS1	LncRNADisease v2.0	7	GAS5	Lnc2Cancer 3.0
8	UCA1	Lnc2Cancer 3.0	8	MALAT1	Lnc2Cancer 3.0
9	KCNQ1OT1	Lnc2Cancer 3.0	9	C1QTNF9B-AS1	Lnc2Cancer 3.0
10	GAS5	Lnc2Cancer 3.0	10	MEG3	Lnc2Cancer 3.0
11	IGF2-AS	Lnc2Cancer 3.0	11	XIST	Lnc2Cancer 3.0
12	HOTAIR	Lnc2Cancer 3.0	12	PVT1	Lnc2Cancer 3.0
13	TUG1	Lnc2Cancer 3.0	13	HOTAIR	Lnc2Cancer 3.0
14	TERC	LncRNADisease v2.0	14	KCNQ1OT1	Lnc2Cancer 3.0
15	CTBP1-AS	Lnc2Cancer 3.0	15	CDKN2B-AS1	LncRNADisease v2.0

Renal cancer is one of the most rapidly growing malignant tumors. Abnormal expression of lncRNAs has been detected in several kinds of renal cancers. It is important to find associations between lncRNAs and renal cancer for cancer prevention, diagnosis, and treatment. The research find that relative level of H19 is significantly higher in clear cell renal carcinoma (ccRCC) compared to the adjacent normal renal tissues. The higher expression of H19 is found in renal cancer cells compared to the nonmalignant renal cells HK-2. So H19 is considered as a potential prognostic indicator and a target for gene therapy of ccRCC [63]. In top 15 results, 93% of lncRNAs are verified to be related to renal cancer (see Table 8). For example, KCQN1OT1 and MALAT-1 are the kidney cancer-associated onco-lncRNAs, and H19 and GAS5 are the kidney cancer-associated tumor suppressive lncRNAs [64].

**Table 8:** Top 15 predicted lncRNAs associated with renal carcinoma

Disease similarity based on gene–gene interaction network			Disease similarity based on disease semantic		
Rank	lncRNA	Evidence	Rank	lncRNA	Evidence
1	TRAF3IP2-AS1	PMID: 33741027	1	TRAF3IP2-AS1	PMID: 33741027
2	H19	LncRNADisease v2.0	2	DLX6-AS1	Lnc2Cancer 3.0
3	XIST	Lnc2Cancer 3.0	3	SNHG11	PMID: 32126023
4	CDKN2B-AS1	Lnc2Cancer 3.0	4	H19	LncRNADisease v2.0
5	MALAT1	Lnc2Cancer 3.0	5	MALAT1	Lnc2Cancer 3.0
6	MIAT	PMID: 30041179	6	CDKN2B-AS1	Lnc2Cancer 3.0
7	UCA1	Lnc2Cancer 3.0	7	XIST	Lnc2Cancer 3.0
8	DRAIC	LncRNADisease v2.0	8	MIAT	PMID: 30041179
9	MIR17HG	PMID: 24511118	9	GAS5	Lnc2Cancer 3.0
10	MEG3	Lnc2Cancer 3.0	10	MEG3	Lnc2Cancer 3.0
11	KCNQ1OT1	LncRNADisease v2.0	11	NEAT1	Lnc2Cancer 3.0
12	NEAT1	Lnc2Cancer 3.0	12	KCNQ1OT1	LncRNADisease v2.0
13	TUG1	Lnc2Cancer 3.0	13	UCA1	Lnc2Cancer 3.0
14	PCAT29	LncRNADisease v2.0	14	LSINCT5	unconfirmed
15	MINA	unconfirmed	15	MIR17HG	PMID: 24511118

The experimental results show that the prediction results using the disease similarity based on gene–gene interaction network are more accurate than other results using the disease similarity based on disease semantic.

## Conclusions

Predicting disease-related lncRNAs will help people understand the underlying pathogenesis of diseases. To overcome the time-consuming and expensive shortcomings of experimental methods, researchers have focused on identifying lncRNA-disease potential association by computational methods.

In this paper, we propose an effective LDA prediction method using graph-level graph attention network called gGATLDA. We firstly extract enclosing subgraphs of lncRNA-disease pairs from lncRNA-disease bipartite graph. Next, we compute lncRNA/disease similarity to construct the features of lncRNA/disease nodes in subgraphs. Finally, graph attention network is used to classify lncRNA-disease pairs into true pairs and false pairs according the subgraphs and feature vectors. Three datasets are used to verify the performance of gGATLDA. We compare gGATLDA with several state-of-the-art methods. The experimental results show that our method gGATLDA can achieve higher values of AUC and AUPR. Furthermore, case study also show that our method can accurately predict LDAs. In the future, we will further improve the prediction performance of gGATLDA by the following aspects. Firstly, we will study better selecting negative sample method to avoid false negative caused by random selection. Secondly, lncRNA similarity and disease similarity are important to improve the prediction performance. At present, most models only use lncRNA-disease functional similarity based on lncRNA-disease interaction. In addition, there are lncRNA/disease similarities based on other different biological data sources, such as lncRNA expression based functional similarity, GO term based lncRNA functional similarity, lncRNA-disease association based functional similarity and miRNA/mRNA-lncRNA interaction based functional similarity. Each similarity has its own strengths and weaknesses [65]. We will study methods for integrating different functional similarities. Lastly, we will extend our method to predict potential interaction relationship in other biologic interaction networks.

Moreover, the advancement of miRNA-disease association prediction can provide valuable reference for LDAs prediction. For example, Chen et al. [66] presented a model of inductive matrix completion for miRNA-disease association prediction. This method based on matrix completion had been successfully applied to LDA prediction [48]. However, the miRNA-disease prediction methods based on matrix decomposition and heterogeneous graph inference had been not used to LDA prediction [67]. Therefore, we will study how can more accurate predict lncRNA-disease potential associations in the future work by referencing some important computational models in literate [68].

## Datasets and methods

### Datasets

**Fig. 6:**
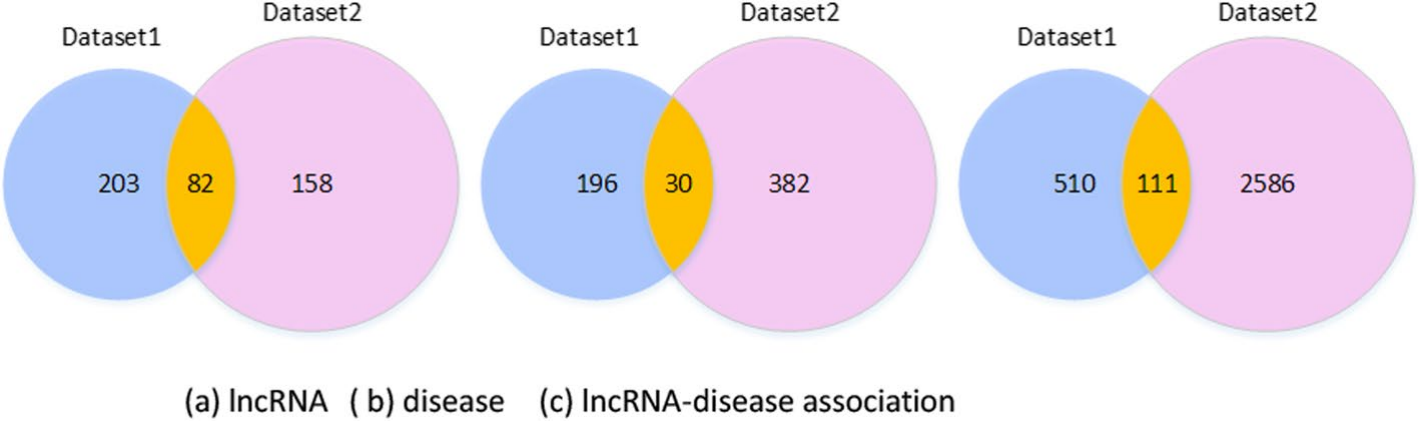
Venn diagrams of the two datasets

In order to experimentally verify the advantages of the method gGATLDA, we use two benchmark lncRNA-disease datasets: one dataset contains fewer known LDAs and another dataset contains more known LDAs. We download the Dataset1 from the lncRNADisease established in 2015, which includes 621 associations between 256 lncRNAs and 189 diseases. The Dataset2 in literate [47] is downloaded from http://mlda.swu.edu.cn/codes.php?name=MFLDA, which includes 2697 associations between 240 lncRNAs and 412 diseases. Dataset density represents the proportion of known associations among all in the dataset. The density of Dataset1 and Dataset2 are 0.96% and 2.73% respectively. We obtain Dataset3 by merging the two datasets Dataset1 and Dataset2. The overlap of Dataset1 and Dataset2 are shown in Fig. 6. We remove all repeated lncRNAs and diseases in Dataset1 and Dataset2. Finally, we obtain 3207 known associations between 443 lncRNAs and 608 diseases. The three benchmark datasets are shown in Table 9.

**Table 9:** Three benchmark datasets

Datasets	lncRNAs	Diseases	Associations
Dataset1	285	226	621
Dataset2	240	412	2697
Dataset3	443	608	3207

We construct an adjacency matrix $A\in {\mathbb{R}}^{L\times D}$ to represent the association pairs between *L* lncRNAs and *D* diseases, where $A\left(l,d\right)=1$ if there is an experimentally verified association between lncRNA $l$ and disease $d$, otherwise $A\left(l,d\right)=0$.

1 $$A\left(l,d\right)=\left\{ \begin{array}{c}1, if\ lncRNA\ l\ associated\ with\ disease\ d \\ 0, otherwise\end{array}\right.$$

### Gaussian interaction profile kernel similarity of lncRNAs

Gaussian kernel function has been used to effectively measure lncRNA similarity [48]. Let the lncRNA similarity matrix be ${S}_{lnc}\in {\mathbb{R}}^{L\times L}$. The Gaussian interaction profile kernel similarity ${S}_{lnc}\left({l}_{i},{l}_{j}\right)$ between lncRNA ${l}_{i}$ and ${l}_{j}$ can be calculated as follows:

2 $${S}_{lnc}\left({l}_{i},{l}_{j}\right)=\mathrm{exp}\left(-{\beta }_{l}{\Vert IP\left({l}_{i}\right)-IP\left({l}_{j}\right)\Vert }^{2}\right)$$

where the *i*th row $IP({l}_{i})$ of the lncRNA-disease association matrix is a binary vector, which represents whether lncRNA ${l}_{i}$ is associated with each disease, *i* = 1, 2 , … , *L*. The normalized bandwidth ${\beta }_{l}$ is calculated by the average number of diseases associated with each lncRNA, its formula are as follows:

3 $${\beta }_{l}=\frac{1}{L}\sum_{i=1}^{L}{\Vert IP\left({l}_{i}\right)\Vert }^{2})$$

### Disease similarity computation based on gene–gene interaction network

The network distance between two disease modules indicates their pathobiological and clinical similarity. If two disease modules are topologically separated in the network, they are considered as pathobiologically distinct. If two disease modules are topologically overlapped, the magnitude of the overlap is indicative of their biological relationship. The higher the overlap degree, the more significant pathobiological similarity between the two disease modules [69]. We propose a new disease similarity computation based on gene–gene interaction network. We define a set of all genes related to a disease as a disease module, and measure the disease similarity by distance between two disease modules in the gene interaction network. The shorter their distance, the more similar the two diseases. The calculation of disease similarity based on gene–gene interaction network are mainly described as follows:

(1)We download the two datasets, one is disease-gene associations from the database DisGeNET at https://www.disgenet.org/ [70] and another one is gene–gene interaction network at https://science.sciencemag.org/content/suppl/2015/02/18/347.6224.1257601.DC1 [69]. We unify the name of diseases of synonymous but different terms in the disease-gene database, and retain the disease-gene association data of those diseases in the benchmark dataset.

(2)For any two diseases, we solve the gene sets associating with diseases ${d}_{i}$ and ${d}_{j}$ respectively according to the disease-gene association network. Let the gene set related to disease ${d}_{i}$ be *A* and the gene set related to disease ${d}_{j}$ be *B*, the mean shortest distance ${S}_{AB}$ between gene sets *A* and *B* is calculated as follows:

4 $${S}_{AB}={d}_{AB}-\frac{({d}_{AA}+{d}_{BB})}{2}$$

where ${d}_{AA}$ is the mean shortest distance of distances among all gene–gene pairs in gene set *A*, ${d}_{BB}$ is the mean shortest distance of distances among all gene–gene pairs in gene set *B*, and ${d}_{AB}$ is the mean shortest one of distances between gene sets *A* and *B*.

(3) The larger the *S*_*AB*_, the greater separation between the two gene sets *A* and *B* associated with disease ${d}_{i}$ and disease ${d}_{j}$ respectively, which means the higher similarity between diseases ${d}_{i}$ and ${d}_{j}$. On the other hand, the smaller the *S*_*AB*_, the larger overlap between the two gene sets *A* and *B* associated with disease ${d}_{i}$ and disease ${d}_{j}$ respectively, which means the lower similarity between diseases ${d}_{i}$ and ${d}_{j}$. The similarity ${S}_{dis}\left({d}_{i},{d}_{j}\right)$ between diseases ${d}_{i}$ and ${d}_{j}$ based on gene–gene interaction network is calculated as follows:

5 $${S}_{dis}\left({d}_{i},{d}_{j}\right)=1-\frac{{S}_{AB}-\mathrm{min}\left({S}_{AB}\right)}{\mathrm{max}\left({S}_{AB}\right)}$$

## Methods

**Fig. 7:**
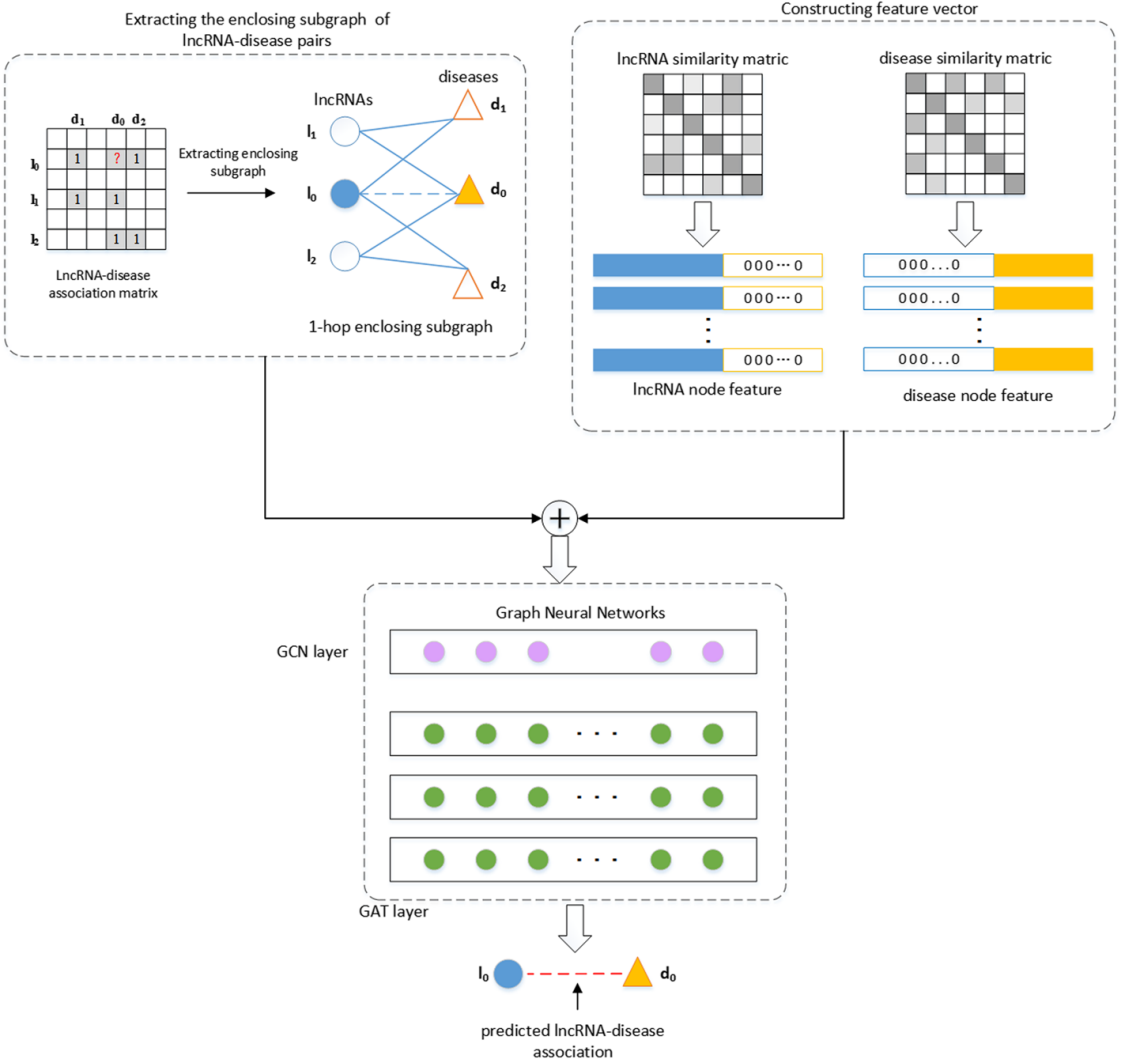
Procedure of the method gGATLDA

In this paper, we propose a new lncRNA-disease association prediction method based on graph-level graph attention network called gGATLDA. As illustrated in Fig. 7, the gGATLDA consists of the following three major steps. Firstly, the enclosing subgraphs of lncRNA-disease pairs are extracted according to lncRNA-disease bipartite graph. Secondly, feature vectors of lncRNA-disease pairs are constructed according to Gaussian interaction profile kernel lncRNA similarities and gene interaction network-based disease similarities. Finally, the subgraphs and feature vectors of the lncRNA-disease pairs are used as the inputs to train the graph attention network model, a probability score of each lncRNA-disease pair is obtained, and the potential LDAs are predicted by ranking these probability scores.

### Extracting the enclosing subgraphs

For the known LDAs matrix *A*, its corresponding bipartite graph *G* can be constructed. If there is an association between lncRNA *l* and disease *d*, there is an edge between nodes *l* and *d* in *G*, otherwise there is no an edge between nodes *l* and *d*. The *h*-hop enclosing subgraph *G*_1_(*V*_1_,*E*_1_) of each lncRNA-disease pair (*l,d*) is defined as the following: *V*_1_ is node set including nodes *l* and *d*, as well as their *h*-hop neighbor nodes, ${E}_{1}$ is edge set, $\forall (u,v)\in {E}_{1}$, there must be $u,v\in {V}_{1}$.

The previous LDAs prediction method based on GNN used node embedding as input to GNN. Considering that local subgraphs can contain richer graph patterns, we extract the *h*-hop enclosing subgraphs of each lncRNA-disease node pair, and use them as the input to train GAT-based model for improving prediction performance.

### Node labeling

Each node in the subgraph can be labeled to distinguish its role [71]. We use 0 and 1 to label the target lncRNA node and target disease node respectively. For other nodes in subgraph, if it is an lncRNA-type node, we will label it as 2*i*; if it is a disease-type node, we will label it as 2*i* + 1, where *i* is a number in the *i-*th hop neighbor of the target node.

### Constructing feature vectors for lncRNAs/diseases

The feature vector for each lncRNA node is constructed based on lncRNA similarity, and the feature vector for each disease node is constructed based on disease similarity. The feature vectors are used as node attribute of subgraph. Let lncRNA similarity matrix be ${S}_{lnc}\in {\mathbb{R}}^{L\times L}$ and disease similarity matrix be ${S}_{dis}\in {\mathbb{R}}^{D\times D}$, where *L* and *D* is the number of lncRNAs and diseases respectively, we construct the lncRNA feature matrix ${F}_{lnc}\in {\mathbb{R}}^{L\times \left(L+D+K\right)}$ and the disease feature matrix ${F}_{dis}\in {\mathbb{R}}^{D\times (L+D+K)}$. In order to make the feature vector dimension of lncRNA the same as that of disease, the feature vector of lncRNA *l* is ${f}_{l}=\{{f}_{l1},{f}_{l2},{f}_{l3},\dots ,{f}_{{l}_{m}}, 0, 0,\dots ,0,{b}_{1},{b}_{2},\dots ,{b}_{K}\}$, and the feature vector of disease *d* is ${f}_{d}=\{0, 0,\dots ,0,{f}_{d1},{f}_{d2},{f}_{d3},\dots ,{f}_{{d}_{n}},{b}_{1},{b}_{2},\dots ,{b}_{K}\}$, where $1\le m\le L$, $1\le n\le D$, *b*_*j*_ is the *k*-bit one-hot code of the node label, *j* = 1,2,…,*K*.

### The model based on graph neural network

We employ a stacked graph neural network layers as the classifier for predicting LDAs. The *h*-hop enclosing subgraph *G*_1_ for lncRNA *l* and disease *d* and feature vectors of each node in *G*_1_ are fed into prediction model. The model is trained to obtain prediction score between lncRNA *l* and disease *d*.

As shown in Fig. 7, our model includes a single graph convolutional network  (GCN) layer and multilayer graph attention network (GAT) layer. Here, we first leverage GCN to learn graph patterns by aggregating representations of their neighborhood nodes to obtain lncRNA/disease latent features. The first layer, i.e., the GCN layer, is formulated as follows:

6 $${x}_{i}^{1}=\sum_{j\in \mathcal{N}\left(i\right)\cup \{i\}}\frac{1}{\sqrt{\mathrm{deg}\left(i\right)}\bullet \sqrt{\mathrm{deg}\left(j\right)}}({W}^{1}{x}_{j}^{0})$$

where ${x}_{j}^{0}$ denotes the feature vector of node *j* in layer 0 (input layer), $\mathcal{N}\left(i\right)$ denotes the set of all neighbor nodes of node *i*, $\mathrm{deg}\left(i\right)$ denotes the degree of node *i*, and ${W}^{1}$ denotes the parameter matrix to be learned of the GCN layer.

Most of the GNNs use a messaging-passing scheme in which the embedding of a node is iteratively updated by aggregating the information from its neighbors [72]. To assign learnable weights in the aggregation, GNNs incorporate the attention mechanism. When aggregating neighbor embedding, the characteristics of neighbor nodes are weighted by attention coefficients between current node and its neighbors, such that GNNs can pay more attention to important nodes to reduce the impact of edge noise. Therefore, after the first GCN layer, we stack multi-layer graph attention layer. The output feature ${h}_{i}^{(l+1)}$ of the *l* + 1th layer is calculated as follows:

7 $${e}_{ij}^{l}=a(W{h}_{i}^{\left(l\right)},W{h}_{j}^{(l)})$$

8 $${\alpha }_{ij}^{l}={softmax}_{i}({e}_{ij}^{l})$$

9 $${h}_{i}^{(l+1)}=\upsigma (\sum_{j\in \mathcal{N}(i)}{\alpha }_{ij}{W}^{(l)}{h}_{j}^{(l)})$$

where $a$ is a function for calculating the correlation between two nodes, ${e}_{ij }^{l}$ is the original attention coefficient between node *i* and node *j* in the *l*-th layer, ${\alpha }_{ij}^{l}$ is the attention weight by softmax function, ${h}_{i}^{(l+1)}$ is the representation of node *i* in the *l* + 1-th layer, $\upsigma$ denotes the non-liner activation function. Here we choose ELU as activation function.

For the output of the *L*th GAT layer, we concatenate the final representations of the target lncRNA and disease as graph representation ${g}_{i}$:

10 $${g}_{i}=\mathrm{concat}({h}_{lnc},{h}_{dis})$$

Finally, for the graph representation ${g}_{i}$, we use Softmax function to obtain the prediction likelihood ${y}_{i}^{^{\prime}}$:

11 $${y}_{i}^{^{\prime}}=\mathrm{softmax}\left({g}_{i}\right)=\frac{{e}^{{g}_{i}}}{{\sum }_{j=1}^{n}{e}^{{g}_{i}}},i=\mathrm{1,2},\dots ,n$$

The weights ${W}^{(l)}$ are trained to minimize the loss function:

12 $$\mathcal{L}=-\sum_{k=1}^{N}({y}_{i}*log{y}_{i}^{^{\prime}})$$

where ${y}_{i}$ represents the real value, ${y}_{i}^{^{\prime}}$ is the predicted value.

Based on the above work, we presented a graph-level graph attention network based LDA prediction algorithm called gGATLDA.
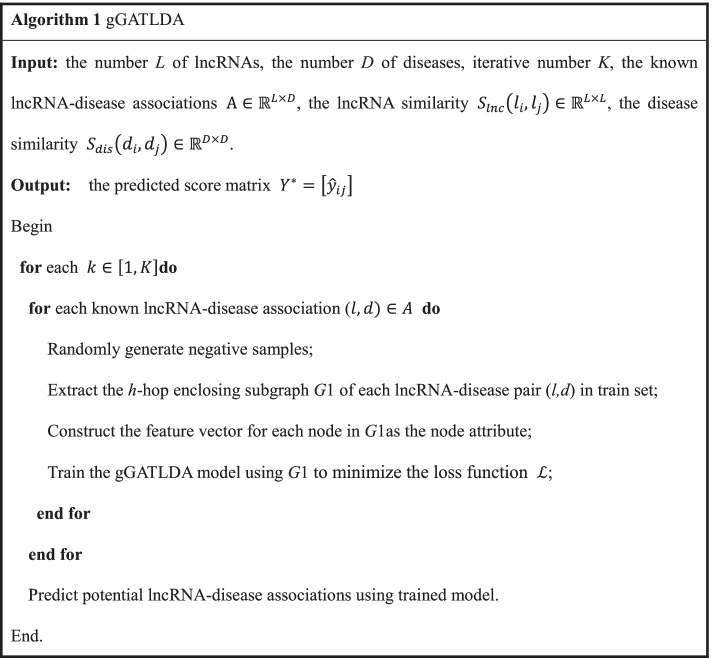


## Data Availability

All the data used are collected from the public datasets below. The Dataset1can be downloaded from the lncRNADisease established in 2015 (http://www.cuilab.cn/lncrnadisease). The Dataset2 can be downloaded from http://mlda.swu.edu.cn/codes.php?name=MFLDA. The source code is available at https://github.com/LiWangG/gGATLDA.

## Abbreviations

gGATLDA: LncRNA-disease association prediction method based on graph-level graph attention network
GCN: Graph convolutional network
GAT: Graph attention network
ROC: Receiver operating characteristic
AUC: Areas under ROC curve
AUPR: Areas under precision-recall curve
